# IQ as a predictor of functional outcome in schizophrenia: A longitudinal, four-year study of first-episode psychosis

**DOI:** 10.1016/j.schres.2008.08.014

**Published:** 2009-01

**Authors:** Verity C. Leeson, Thomas R.E. Barnes, Sam B. Hutton, Maria A. Ron, Eileen M. Joyce

**Affiliations:** a Department of Psychological Medicine, Imperial College London, Charing Cross Campus, St. Dunstan’s Road, London W6 8RP, UK; b Department of Psychology, University of Sussex, Brighton BN1 9QG, UK; c Institute of Neurology, University College London, The National Hospital for Neurology and Neurosurgery, Queen Square, London WC1N 3BG, UK

**Keywords:** Psychosis, Schizophrenia, IQ, Cognition, Outcome

## Abstract

Studies of established schizophrenia have consistently found that cognitive function predicts social and clinical outcomes. The findings from first-episode studies have been more variable, with only some studies reporting predictive relationships. We tested the possibility that an index of general cognitive ability, IQ, may be a more sensitive and reliable predictor of outcome in first-episode schizophrenia than specific measures of memory and executive function. Fifty-four patients with first-episode schizophrenia or schizoaffective disorder were assessed for cognitive and social function as well as symptoms at three time points over the four years following first presentation of their psychotic illness. Regression analyses were performed to determine whether IQ and specific neuropsychological measures at first episode and one-year follow-up predicted four-year social function and residual symptoms. The effects of premorbid and concurrent IQ on outcome were also assessed. Premorbid IQ and IQ at each assessment significantly predicted social function at four-year follow-up. This relationship remained significant after the social function or symptom scores at first presentation were accounted for in the regression. Specific measures predicted certain domains of social function, but these were weaker and less consistent than IQ. The predictive values of cognition on residual symptoms were less strong; the most consistent finding was a relationship between IQ and the negative syndrome. This study suggests that early in the course of schizophrenia, general cognitive ability, as measured by IQ, is a more sensitive and reliable predictor of functional outcome than measures of specific ability.

## 1 Introduction

Schizophrenia is a complex neurodevelopmental disorder with cognitive impairment as a core feature (Joyce and Huddy, 2004). The hypothesis that poor clinical and social outcome is related to the degree of impairment in cognitive function stems from systematic reviews of cross-sectional studies of patients with established schizophrenia (Green, 1996; Green et al., 2000). The results of longitudinal studies, which assessed patients entering rehabilitation schemes or continuing with usual treatment, support this view. With only one exception (Norman et al., 1999), these studies have found that at least some aspects of cognitive function predict outcome between 6 months and 15 years later (Brekke et al., 2005; Bryson and Bell, 2003; Dickerson et al., 1999; Fujii and Wylie, 2003; Gold, 2004; Jaeger et al., 2003; Prouteau et al., 2005; Smith et al., 2002; Velligan et al., 2000; Woonings et al., 2003).

An understanding of the predictors of outcome at illness onset is particularly important because of the potential to shape early interventions. However, the findings of longitudinal first-episode studies are less consistent than those in patients with established schizophrenia. Whereas some studies assessing outcome up to 10 years after illness onset have found significant cognitive predictors at the time of the first episode (Holthausen et al., 2007; Milev et al., 2005), others have not (Malla et al., 2002; Stirling et al., 2003; Verdoux et al., 2002). Some prospective studies have measured cognitive function at different time points following psychosis onset, as well as outcome, and found that concurrent but not previous cognition was associated with social function (Addington et al., 2005; Malla et al., 2002; Stirling et al., 2003). These findings suggest that neuropsychological measures in the early stages of schizophrenia may not be reliable predictors of outcome.

Most studies in this area have examined the impact of specific domains of cognitive function on outcome, such as memory, attention and executive function. Few have incorporated IQ as a measure of general ability (Carlsson et al., 2006; Fujii and Wylie, 2003; Gold et al., 2002; van Winkel et al., 2007). This is surprising since IQ decrements are widely reported and premorbid IQ is regarded as a risk factor for the subsequent development of schizophrenia (Badcock et al., 2005; David et al., 1997; Fuller et al., 2002; Jones et al., 1994; Joyce et al., 2005; Kremen et al., 1998; Weickert et al., 2000). Gold et al. (2002) in a study of established schizophrenia found that out of a wide variety of measures, IQ had the highest correlation with total hours worked over the subsequent 12 months. First-episode studies have also found that premorbid IQ and IQ at first episode are significant predictors of outcome (Carlsson et al., 2006; van Winkel et al., 2007).

In the current study, we examined the effect of premorbid IQ, and IQ at three assessment periods – first episode, one and four-year follow-up – on social and clinical outcome at the four-year follow-up assessment. We also examined the effect of more specific measures of neuropsychological performance in the domains of memory and executive function. We were able to test the stability of neuropsychological measures as predictors of subsequent outcome and whether previous or concurrent measures and specific or general abilities were better in this regard.

## 2 Materials and methods

### 2.1 Subjects

The patients were recruited as part of a longitudinal study of first-episode psychosis in West London and were eligible if aged between 16 and 55 years, presenting from the community to mental health services with a psychotic illness for the first time and had no more than 12 weeks cumulative exposure to antipsychotic medication. Fifty-four patients (43 male, 10 female) completed 3 assessments: at presentation with their first psychotic episode and a mean of 17 months (one-year follow-up) and 52 months (four-year follow-up) later. Fifty-two had a DSMIV diagnosis of schizophrenia and two of schizoaffective disorder at the four-year follow-up assessment. At the time of initial assessment, six were not being prescribed antipsychotic medication, 27 were prescribed first-generation and 19 second-generation antipsychotics, and two were prescribed a combination of both.

### 2.2 Clinical assessments

Patients were assessed with the Scales for the Assessment of Positive and Negative Symptoms (SAPS: Andreasen, 1984; SANS: Andreasen, 1983). Scores for the three symptom derived syndromes of schizophrenia (positive, negative, disorganisation; Liddle and Barnes, 1990) were calculated for each patient. Social function was assessed with the Social Function Scale (SFS), a 79-item, self-report scale, which Birchwood et al. (1990) showed to be a reliable, valid and sensitive measure of social functioning in individuals with schizophrenia. Individuals rate their abilities in seven areas of social function (activation-engagement, interpersonal communication, frequency of activities of daily living, competence at activities of daily living, participation in social activities, participation in recreational activities, and employment or occupational activity) to provide subscale scores and a global score.

### 2.3 Neuropsychological assessments

Premorbid IQ was estimated using the Revised National Adult Reading Test (NART: Nelson and Willison, 1991). Two patients did not undergo assessment with the NART as they were dyslexic. Current IQ was calculated from four subtests of the Wechsler Adult Intelligence Scale-Revised (WAIS-R: Wechsler, 1997; Missar et al., 1994). Memory and executive functions were measured with computerised tests from the Cambridge Automated Neuropsychological Test Battery (CANTAB: Sahakian and Owen, 1992) as follows: *Pattern recognition memory* — 24 single abstract patterns are shown sequentially followed by forced choice recognition trials. Total correct was used as an index of recognition memory; *Spatial working memory* — this is a self-ordered search task which measures the ability to remember the location of previously found ‘tokens’ while searching for new tokens. An error occurs when a participant returns to the location where a token has already been found. Total errors were used as an index of the ability to manipulate information in working memory; *Spatial Span* — this measures the ability to remember the order of sequences of coloured squares on the screen presented in increasing numbers. The highest number recalled correctly was used as an index of working memory span; *Stockings of Cambridge* — this is an analogue of the Tower of London (Shallice, 1982). On trials which vary in difficulty, subjects move coloured ‘balls’ in an arrangement displayed on the screen to match a goal arrangement. Planning was indexed by the number of problems solved in the minimum number of moves possible. Response times are recorded during planning trials and also during yoked control trials that mimic the exact sequence of moves generated by the subject during planning. An index of information processing speed or ‘thinking time’ was derived by subtracting the time taken to initiate the first move in the control trial from that in the equivalent planning trial. *Attentional set shifting* — this test disambiguates the psychological processes contributing to performance of the Wisconsin Card Sorting Test. Participants are required to deduce a rule which governs the ‘correct’ response in a series of visual discriminations. Once correct responding is established, the ‘rule’ required for correct responding is changed. We used the number of errors made on early stages of the task as an index of rule learning. At a later stage the features governing the rule are changed and participants are required to switch attentional set for correct responding. Errors at this stage were used as an index set-shifting ability.

In summary, the neuropsychological measures used as predictors of four-year social function and symptoms were: IQ, working memory span and manipulation, recognition memory, rule learning, set shifting, planning and thinking time.

### 2.4 Statistical analysis

Seven percent of the neuropsychological data points were missing, accounted for by non-completion of test procedures. Missing data analyses using regression, based on the repeated measures scores for each given test variable, were used to impute these missing values.

At initial assessment, there was a sufficient range of values for each clinical syndrome score to allow for the variable to be continuous. However, at one-year and four-year follow-up, the scores were non-normally distributed. For these two time points the scores were dichotomised for each syndrome as *present* or *absent*. Thinking time was transformed to log_10_ in order to return the distribution to normal.

To determine whether cognitive functions predicted later outcome, hierarchical regression analyses were performed with four-year follow-up clinical or social function measures as the dependent variable. The corresponding measure at initial assessment was entered as a predictor in the first block and all neuropsychological measures were entered as potential predictors in the second block using stepwise entry criteria. These regression analyses were linear for social function measures and logistic for syndrome scores. For all stepwise regression analyses the criterion for entry was *p* < 0.10. In separate regressions, we examined the effect of initial and one-year follow-up neuropsychological measures on four-year social function or syndrome scores.

## 3 Results

Table 1 shows the mean performance of the patient group on all cognitive measures at each assessment. Pearson’s correlations for the cognitive test scores at initial assessment showed that current IQ was highly associated with recognition memory (*r* = 0.44, *p* < 0.001), working memory manipulation (*r* = 0.45, *p* < 0.001) and working memory span (*r* = 0.44, *p* < 0.001) but not with the remaining variables (range of *r*s = 0.05–0.22). At one-year follow-up, current IQ was significantly correlated with recognition memory (*r* = 0.47, *p* < 0.001), working memory manipulation (*r* = 0.37, *p* = 0.005), working memory span (*r* = 0.40, *p* = 0.003), set shifting (*r* = 0.46, *p* < 0.001), rule learning (*r* = 0.29, *p* = 0.033), planning (*r* = 0.30, *p* = 0.026) but not thinking time (*r* = 0.01, *p* = 0.921).

The results of the main regression analyses are shown in Table 2. After entry of initial SFS score, a significant amount (24%) of the remaining variance in four-year SFS score was explained by IQ and thinking time at the initial assessment. IQ and thinking time predicted the SFS subscale score of activation-engagement, explaining 23% of the variance. IQ alone predicted frequency of activities of daily living (19% variance) and occupational activity (16% variance). Thirty-two percent of the variance in interpersonal communication was explained by a number of neuropsychological measures including IQ and thinking time. When neuropsychological measures at one-year follow-up were used, IQ was the sole predictor of global social function (12% variance) and the main predictor of frequency of activities of daily living and occupational activity.

There were no consistent predictors of symptoms at four-year follow-up. IQ, thinking time and recognition memory at initial assessment together predicted 50% of the variance in the negative syndrome, whereas thinking time alone predicted the positive syndrome (14% variance) and recognition memory alone the disorganisation syndrome (25% variance). One-year follow-up measures showed a different predictive pattern, with set-shifting predicting negative symptoms (10% variance) and planning predicting disorganisation (15% variance).

We also examined the predictive values of premorbid and concurrent IQ using regression analysis, and found that both variables significantly contributed to global four-year social function (premorbid IQ: *r*^2^_adj_ = 0.08, *t* = 2.40, *p* = 0.021; four-year IQ: *r*^2^_adj_ = 0.12, *t* = 4.72, *p* = 0.014). The presence of the negative syndrome at four-year follow-up was also predicted by four-year IQ (Wald = 5.14, *p* = 0.023, odds ratio = 0.95 Nagelkerke *r*^2^ = 0.17) but not by premorbid IQ (Wald = 0.50, *p* = 0.478, Nagelkerke *r*^2^ = 0.02). Verbal and performance IQ were additionally examined independently to assess the contribution of verbal and non-verbal skills. Using a prorated score based on the two subtests from either the performance or verbal tests at initial assessment, both were significant predictors of global four-year social function (verbal IQ: *r*^2^_adj_ = 0.19, *t* = 3.38, *p* = 0.002; performance IQ: *r*^2^_adj_ = 0.13, *t* = 2.84, *p* = 0.007).

Finally, because other studies have reported that a proportion of the variance in follow-up social functioning is accounted for by symptoms measured at an earlier time point, we tested whether including symptom variables in the model prior to testing for the relative contribution of neuropsychological measures had an effect. We performed an hierarchical linear regression with four-year follow-up SFS scale score as the dependent variable, all three syndrome scores at first episode entered into the first block as predictors and current IQ at first episode entered into the second block. The results showed that IQ continued to explain a significant additional proportion of the variance once the influence of syndrome scores was accounted for (*r*^2^_adj_ = 0.13, *t* = 3.08, *p* = 0.003). A similar analysis with age at illness onset and sex entered in the first block also showed IQ to remain a significant predictor (*r*^2^_adj_ = 0.30, *t* = 4.180, *p* < 0.001).

## 4 Discussion

One of the aims of this study was to determine whether general or specific aspects of cognitive function early in the course of schizophrenia predicted later social and clinical outcomes. We examined the effect of IQ and seven indices of memory and executive function on social function a mean of four years after first presentation with psychosis. Social function was measured with the SFS which assesses seven domains of social activity and also provides a global index of social function (Birchwood et al., 1990). Of the cognitive measures used, IQ and thinking time at illness onset were the only predictors of global social function and these accounted for 24% of the variance. These two measures also uniquely predicted the domain of activation-engagement and contributed to the prediction of interpersonal communication. IQ alone predicted the frequency of activities of daily living and occupational activity. Although more specific measures of cognitive function also made contributions to various social domains, these associations were weaker and less consistent than those with IQ. We checked that this was not due to the fact that the memory and executive measures were all non-verbal by examining prorated verbal and performance IQ scores independently. Both were strong predictors of four-year global social function indicating that the IQ effect reflected general ability incorporating both verbal and non-verbal skills.

In relation to first-episode psychosis, Addington et al. (2005) have argued that cognitive function after one year is a more appropriate baseline from which to assess predictors of outcome, as any fluctuations due to a disturbed mental state should be less evident at this time. Accordingly, we substituted the same variables measured at one-year follow-up for those at first episode and repeated the analysis. IQ at one-year follow-up predicted global social function and contributed to two of the four social function domains predicted by IQ at illness presentation, namely frequency of activities of daily living and occupational activity. Of the specific cognitive functions, no single variable more reliably or more strongly predicted social outcome than IQ, although working memory manipulation predicted three of the seven domains of social function on this occasion.

Several studies have failed to find any association between cognitive function and social function assessed at different times over the first 10 years of psychotic illness (Malla et al., 2002; Stirling et al., 2003; Verdoux et al., 2002). Others have found associations between specific aspects of cognition and social function (Holthausen et al., 2003; Milev et al., 2005). Studies which combined different neuropsychological measures to derive a composite cognitive score are also contradictory in their findings (Addington et al., 2005; Holthausen et al., 2007; Robinson et al., 2004; Stirling et al., 2003). In our study, the predictive values of individual measures of cognitive function were generally weak and there were changes in the measures predicting specific domains of social function between first assessment and one-year follow-up. These findings suggest that specific neuropsychological domain measures in the early stages of schizophrenia are unreliable predictors of later social outcome.

Few first-episode studies have assessed the impact of IQ on later social function (Carlsson et al., 2006; Malla et al., 2002; van Winkel et al., 2007), and to our knowledge ours is the only one to examine the predictive value of IQ and specific cognitive functions at more than one time point following psychosis onset. Malla et al. (2002) and Carlsson et al. (2006), in mixed groups of schizophrenia and non-schizophrenia patients, did not find a predictive relationship between IQ at first episode and social function one year later. The study by Carlsson et al. (2006) also assessed three-year outcome and, on this occasion, found that first-episode IQ differentiated those with good and poor social function. Along with our finding that IQ predicted social function at four-year follow-up, this suggests that it is possible to predict medium term social outcome using IQ. However social function in the first year of psychotic illness may well be unstable making it difficult to determine true relationships with any measure of cognition, including IQ.

van Winkel et al. (2007) suggested that IQ at illness onset is an unreliable predictor of social outcome and that premorbid IQ and IQ at the time of outcome assessment are better predictors. However, whilst we confirmed that premorbid and concurrent IQ are significant predictors of social function, our results indicate that IQ at first presentation and also at one-year follow-up can be used to predict later social function. Taken together the findings suggest that IQ is both a sensitive cognitive predictor of outcome and a stable trait impacting on function early in the course of schizophrenia.

Previous first-episode studies have found that when both neuropsychological test scores and symptoms were included in regression analyses, the predictive value of various cognitive measures became greatly reduced (Addington et al., 2005; Milev et al., 2005). We examined this possibility by performing a regression analysis which entered initial positive, negative and disorganisation syndrome scores ahead of IQ and found that the predictive effect of IQ was unchanged. Thus the relationship between IQ and later social function did not appear to be a proxy for the association between symptoms and social function; IQ predicted a unique part of the variance in social outcome, regardless of any predictive influence of symptoms on this outcome.

The relationships between neuropsychological performance and the presence of residual symptoms at four-year follow-up were also examined. The most consistent finding was that IQ predicted the presence of the negative syndrome, although this was found only using first assessment and concurrent measures of IQ. Other studies have reported similar relationships. Carlsson et al. (2006) found that IQ predicted negative symptoms at one-year although not three-year follow-up assessment, and Stirling et al. (2003) found that a measure of general cognitive ability that was largely comprised of WAIS subtest measures predicted negative symptom severity 10 years later. Thus, in addition to social function, IQ appears also to be relevant to the persistence or later development of negative symptoms but the findings are less consistent.

The amount of the variance explained by IQ was relatively small, ranging from 12% to 17% for current IQ and 8% for premorbid IQ; only the measure of thinking time added any greater predictive value to the model from the measures we employed. However, the size of our effects is similar to or greater than that reported in other studies of first-episode psychosis using different measures. Further, despite the modest percentage of variance explained, our findings suggest that IQ is a measure which can consistently predict later social and clinical outcome in patients with first-episode schizophrenia and, in this context, is better than measures of specific abilities.

## Role of funding source

This study was funded by the Wellcome Trust. This organisation had no further role in the study design; in the collection, analysis and interpretation of data; in the writing of the report; and in the decision to submit the paper for publication.

## Contributors

EMJ, MAR and TREB designed and set up the study, and wrote the protocol. SBH contributed to the collection and interpretation of data. VL conducted the statistical analysis and wrote the first draft of the manuscript. All authors contributed to and have approved the final manuscript.

## Conflict of interest

All authors declare that they have no conflicts of interest.

## Figures and Tables

**Table 1:** Mean cognitive scores as measured at the three assessments

Measure	First episode score	One-year follow-up score	Four-year follow-up score
Premorbid IQ	100.31 (10.92)		
Current IQ	94.04 (14.86)	96.24 (15.16)	92.35 (24.35)
Recognition memory	20.80 (2.80)	20.71 (3.19)	19.87 (3.28)
Working memory manipulation	29.25 (19.99)	27.07 (5.82)	30.83 (18.56)
Working memory span	5.38 (1.14)	5.82 (1.37)	5.87 (1.38)
Planning	7.47 (2.07)	8.24 (2.13)	8.24 (1.95)
Thinking time	5677 (3221)	5946 (3273)	8216 (5970)
Set shifting	10.78 (10.06)	12.53 (11.09)	9.08 (9.40)
Rule learning	11.18 (9.59)	15.48 (25.17)	7.02 (4.34)

Standard deviations are shown in parentheses.

**Table 2:** Results of linear and logistic regression analyses for four-year follow-up SFS global and domain scores and clinical syndrome scores

Four-year clinical/SFS dependent variable		First-episode neuropsychological measures		One-year follow-up neuropsychological measures	
		Significant cognitive predictors	Model fit	Significant cognitive predictors	Model fit
SFS	Global score	IQ: *t* = 3.43, *p* = 0.001	*r*^2^_adj_ = 0.24	IQ: *t* = 2.73, *p* = 0.009	*r*^2^_adj_ = 0.12
		Thinking time: *t* = − 2.37, *p* = 0.022	*F*_3,48_ = 6.06		*F*_2,48_ = 4.11
			*p* = 0.001		*p* = 0.023
	Activation-engagement	Thinking time: *t* = − 3.15, *p* = 0.003	*r*^2^_adj_ = 0.23	Thinking time: *t* = −2.19, *p* = 0.035	*r*^2^_adj_ = 0.10
		IQ *t* = 2.14, *p* = 0.038	*F*_3,48_ = 5.83		*F*_2,48_ = 3.60
			*p* = 0.002		*p* = 0.035
	Interpersonal communication	Thinking time: *t* = −2.14, *p* = 0.038	*r*^2^_adj_ = 0.32	WM manipulation: *t* = 1.98, *p* = 0.054	*r*^2^_adj_ = 0.08
		Set shifting: *t* = −1.71, *p* = 0.095	*F*_6,48_ = 4.71		*F*_1,48_ = .316
		Recog memory: *t* = 2.00, *p* = 0.052	*p* = 0.001		*p* = 0.052
		WM manipulation: *t* = 2.46, *p* = 0.018			
		IQ: *t* = 2.14, *p* = 0.038			
	Frequency of activities of daily living	IQ: *t* = 3.56, *p* = 0.001	*r*^2^_adj_ = 0.19	IQ: *t* = 3.84, *p* < 0.001	*r*^2^_adj_ = 0.23
			*F*_2,48_ = 6.48	Rule learning: *t* = 2.34, 0.024	*F*_3,48_ = 5.72
			*p* = 0.003		*p* = 0.002
	Competence at activities of daily living	Span: *t* = 2.63, *p* = 0.012	*r*^2^_adj _= 0.09	WM manipulation: *t* = 2.10, *p* = 0.041	*r*^2^_adj _= 0.16
			*F*_2,48 _= 3.47	Set shifting: *t* = 2.46, *p* = 0.018	*F*_*4*__,48_ = 3.33
			*p* = 0.040	WM span: *t* = 1.83, *p* = 0.074	*p* = 0.018
	Participation in recreational activities	Rule learning: *t* = − 1.95, *p* = 0.057	*r*^2^_adj_ = 0.13	None	*r*^2^_adj _= 0.00
		Recognition memory: *t* = 1.90, *p* = 0.064	*F*_3,48 _= 3.31 *p* = 0.029		*F*_2,48 _= 0.31 *p* = 0.578
		
	Participation in social activities	Rule learning: *t* = − 2.04, *p* = 0.047	*r*^2^_adj_ = 0.08	WM manipulation: *t* = 2.38, *p* = 0.022	*r*^2^_adj_ = 0.10
			*F*_2,48_ = 2.99	Planning: *t* = − 1.83, 0.073	*F*_3,48_ = 2.86
			*p* = 0.060		*p* = 0.047
	Occupational activity	IQ: *t* = 3.11, *p* = 0.003	*r*^2^_adj_ = 0.16	IQ: *t* = 3.40, *p* < 0.001	*r*^2^_adj_ = 0.22
			*F*_2,48_ = 5.42	Planning: *t* = − 2.73, *p* = 0.009	*F*_3,48_ = 5.51
			*p* = 0.008		*p* = 0.003
Negative syndrome		IQ: Wald = 4.44, *p* = 0.035, odds ratio = 0.93	Nagelkerke *r*^2^ = 0.50	Set shifting: Wald = 3.83, *p* = 0.05, odds ratio = 1.09	Nagelkerke *r*^2^ = 0.10
		Recognition memory: Wald = 3.98, *p* = 0.046, odds ratio = 0.50			
		Thinking time: Wald = 5.11, *p* = 0.024, odds ratio = 2.12			
Positive syndrome		Thinking time: Wald = 4.58, *p* = 0.032, odds ratio = 1.46	Nagelkerke *r*^2^ = 0.14	None	
Disorganisation syndrome		Recognition memory: Wald = 8.25, *p* = 0.004, odds ratio = 0.70	Nagelkerke *r*^2^ = 0.25	Planning: Wald χ^2^ = 5.16, *p* = 0.023, odds ratio = 0.70	Nagelkerke *r*^2^ = 0.15

Corresponding first episode scores were entered as a predictor in the first block and stepwise entry of individual neuropsychological variables in the second block. Models employing both first episode and one-year follow-up neuropsychological measures are contrasted. WM = working memory.
